# A “twelve-section ultrasonic screening and diagnosis method” and management system for screening and treating neonatal congenital heart disease at the grassroots level in Tang County, Hebei Province, China

**DOI:** 10.1186/s12884-024-06569-x

**Published:** 2024-05-15

**Authors:** Yun Cui, Xin-jian He, Le Wang, Yan-hui Fan, Jiao-yang Chen, Ning Zhao, Shuai Zhang, Lei Liu, Jie Yao, Zhe Ren, Di Fan, Jing Chen, Xinjian He

**Affiliations:** 1 Department of Ultrasound diagnosis, Children’s Hospital of Hebei Province, Hebei Medical University, Shijiazhuang, China; 2 Institute of Pediatrics, Children’s Hospital of Hebei Province, Hebei Medical University, Shijiazhuang, China; 3 Outpatient department, Children’s Hospital of Hebei Province, Hebei Medical University, Shijiazhuang, China

**Keywords:** Neonatal CHD, Prevalence, Screening, Echocardiography, Screening and treatment hierarchical management system

## Abstract

**Background:**

To explore a method for screening and diagnosing neonatal congenital heart disease (CHD) applicable to grassroots level, evaluate the prevalence of CHD, and establish a hierarchical management system for CHD screening and treatment at the grassroots level.

**Methods:**

A total of 24,253 newborns born in Tang County between January 2016 and December 2020 were consecutively enrolled and screened by trained primary physicians via the “twelve-section ultrasonic screening and diagnosis method” (referred to as the “twelve-section method”). Specialized staff from the CHD Screening and Diagnosis Center of Hebei Children’s Hospital regularly visited the local area for definite diagnosis of CHD in newborns who screened positive. Newborns with CHD were managed according to the hierarchical management system.

**Results:**

The centre confirmed that, except for 2 newborns with patent ductus arteriosus missed in the diagnosis of ventricular septal defect combined with severe pulmonary hypertension, newborns with other isolated or concomitant simple CHDs were identified at the grassroots level. The sensitivity, specificity and diagnostic coincidence rate of the twelve-section method for screening complex CHD were 92%, 99.6% and 84%, respectively. A total of 301 children with CHD were identified. The overall CHD prevalence was 12.4‰. According to the hierarchical management system, 113 patients with simple CHD recovered spontaneously during local follow-up, 48 patients continued local follow-up, 106 patients were referred to the centre for surgery (including 17 patients with severe CHD and 89 patients with progressive CHD), 1 patient died without surgery, and 8 patients were lost to follow-up. Eighteen patients with complex CHD were directly referred to the centre for surgery, 3 patients died without surgery, and 4 patients were lost to follow-up. Most patients who received early intervention achieved satisfactory results. The mortality rate of CHD was approximately 28.86 per 100,000 children.

**Conclusions:**

The “twelve-section method” is suitable for screening neonatal CHD at the grassroots level. The establishment of a hierarchical management system for CHD screening and treatment is conducive to the scientific management of CHD, which has important clinical and social significance for early detection, early intervention, reduction in mortality and improvement of the prognosis of complex and severe CHDs.

## Background

Congenital heart disease (CHD) is one of the most common congenital malformations and is one of the major causes of death in infants and young children. [1] Previous studies have shown that the prevalence ranges from 4‰ to 50‰. [2] The prevalence of CHD may be related to the level of prenatal screening, screening population, timing of screening, inclusion criteria for CHD patients, screening methods and other factors. Among CHD patients, those requiring intervention in the neonatal period or early infancy are classified as having severe CHD, with a prevalence of approximately 1–3‰. Early detection and intervention are crucial for improving their prognosis. Since 2008, Norway, Sweden, Germany, the United Kingdom and other Western countries have successively reported large-sample studies that conducted pulse oximetry tests to screen for severe neonatal CHD, with a sensitivity of approximately 75-77.8%. [3–6] The pulse oximetry test has a high detection rate for cyanotic CHD, but it is less sensitive for detecting some CHDs without obvious abnormalities in pulse oximetry (such as left heart obstruction diseases). Since 2011, Professor Huang GY’s team at the paediatric hospital affiliated with Fudan University in China has studied dual-index screening method (i.e., cardiac murmur plus pulse oximetry), which has a sensitivity of approximately 93.2% for severe CHD screening. [7] However, due to the high pulmonary artery pressure in the neonatal period, some murmurs are not obvious in neonates with CHD. In addition, as the haemoglobin concentration is high in neonates, cyanosis is not easily detectable due to hypoxemia, which may lead to misdiagnosis of CHD. Furthermore, it is likely to cause false negatives for left heart obstruction diseases and false positives for pulmonary disease interference. [8] The above methods involve indirect clinical index interpretation and cannot be used to determine the type of CHD. Neonates with positive dual-index screening results must undergo ultrasound examination for definite diagnosis. However, the use of ultrasound screening technology for CHD screening in newborns at the grassroots level has not been standardized and has a low popularity rate and high rates of missed diagnosis and misdiagnosis. Primary ultrasound doctors cannot provide reliable ultrasound diagnoses for neonates who screen positive using the dual-index method. They still need to be referred to the diagnosis and treatment centre for ultrasound diagnosis, with which the hierarchical management and scientific treatment of CHD at the grassroots level cannot be realized. There are many sections of ultrasound accurate diagnosis method from diagnosis and treatment centres. Some of them are nonstandard sections, which are not easy to obtain, and it is difficult for primary physicians to master. Therefore, there is an urgent need for a simple and standardized ultrasound screening and diagnosis method for CHD that is easy to popularize at the grassroots level. Moreover, there are few studies on the prevalence of CHD in Hebei Province, especially at the grassroots level. Considering the above problems, the CHD Screening and Diagnosis Center of Hebei Children’s Hospital (referred to as the centre) collaborated with the China Federation of Social Work and carried out the “Love Action” Neonatal CHD Free Screening Project, which was supported by the Tang County Health Bureau policy and funding from the National Energy Group Public Welfare Foundation. The centre selected Tang County to carry out the pilot study; primary physicians were trained to use the “twelve-section ultrasonic screening and diagnosis method” (referred to as the “twelve-section method”) and conducted neonatal CHD screening after passing the examination. We aimed to explore the reliability and feasibility of this method applied at the grassroots level, improve the detection rate of neonatal CHD, especially complex and severe CHD, evaluate the prevalence of CHD, and investigate a new management mode of CHD screening and treatment.

## Methods

### Research participants and methods

#### Participants

All live newborns born in Tang County, Baoding city, Hebei Province, from January 2016 to December 2020 could receive free echocardiographic screening at Tang County Maternal and Child Health Care Hospital. This study was approved by the Medical Ethics Committee of Children’s Hospital of Hebei Province (ethical lot number 2,019,025).

#### Twelve-section method

**Fig. 1:**
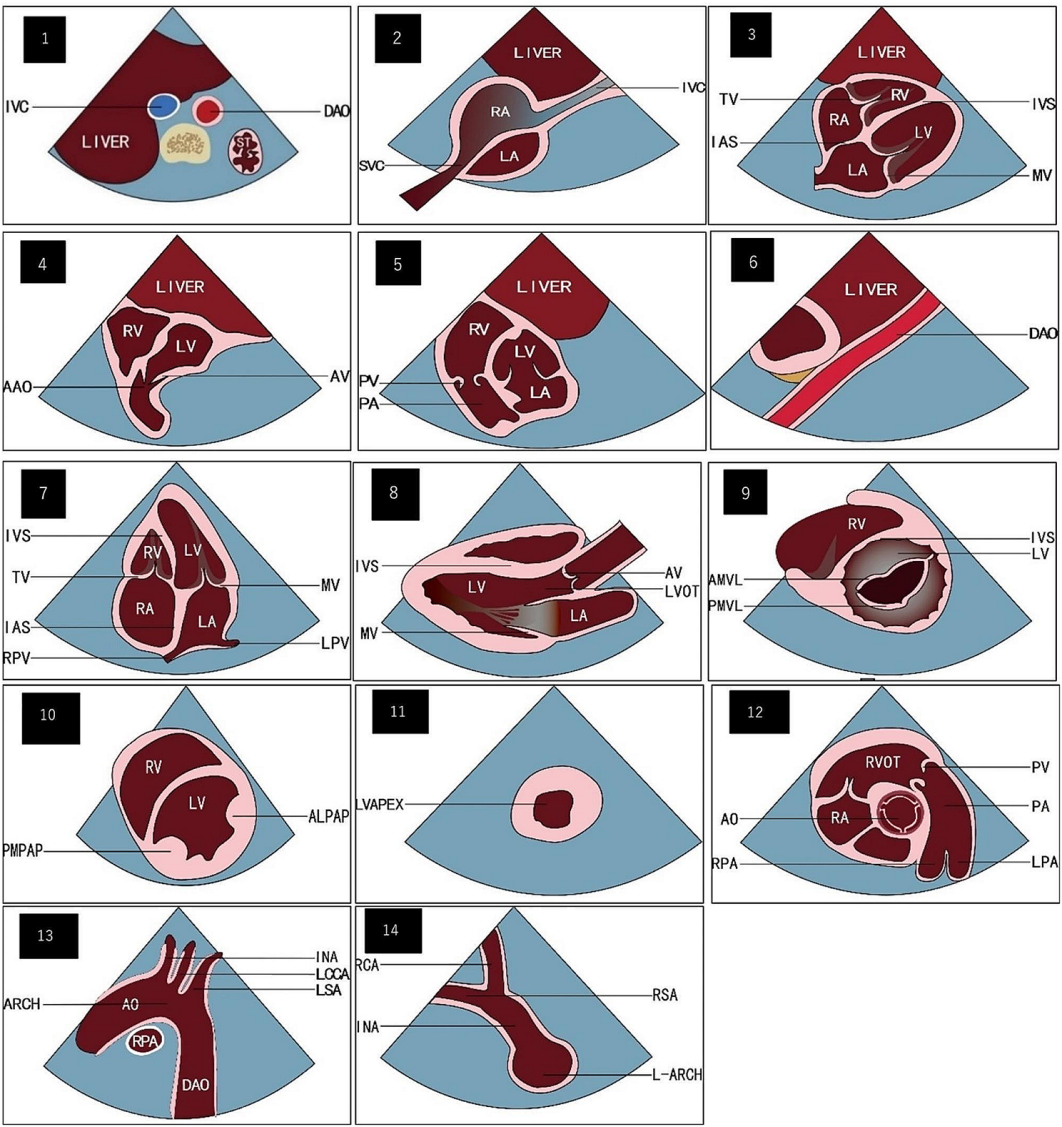
Schematic diagram of the twelve-section method. Figure 1.1 subxiphoid transverse section of abdomen; Fig. 1.2 Subxiphoid section of dual atria; Fig. 1.3 Subxiphoid four-chamber section ; Fig. 1.4 Subxiphoid left ventricular outflow tract section ; Fig. 1.5 Subxiphoid right ventricular outflow tract section; Fig. 1.6 Subxiphoid long axis view of the abdominal aorta; Fig. 1.7 Apical four-chamber section ; Fig. 1.8 Parasternal long axis section of left ventricular; Fig. 1.9–1.11 A series of parasternal left ventricular short axis sections—mitral valve level, papillary muscle level, and cardiac apex level, respectively; Fig. 1.12 Parasternal short axis section of cardiac base great arteries; Fig. 1.13 Long axis section of aortic arch of suprasternal fossa; Fig. 14 Short axis section of aortic arch of suprasternal fossa. AA: abdominal aorta; AAO: ascending aorta; ALPAP: anterolateral papillary muscle; AMVL: anterior mitral leaflet; AV: aortic valve; BM: moderator band; DAO: descending aorta; IAS: interatrial septum; INA: innominate artery; IVC: inferior vena cava; IVS: interventricular septum; LA: left atrium; L-ARCH: left aortic arch; LCCA: left common carotid artery; LPA: left pulmonary artery; LPV: left pulmonary vein; LR: left ventricular; LSCA: left subclavian artery; LVAPEX: left ventricular apex; LVOT: left ventricular outflow; MPA: main pulmonary artery; MV: mitral valve; PA: pulmonary artery; PV: pulmonary valve; RA: right atrium; RCA: right common carotid artery; PMVL: posterior mitral leaflet ; PMPAP: posterior medial papillary muscle; RPA: right pulmonary artery ; RPV: right pulmonary vein; RSA: right subclavian artery; RV: right ventricular; RVOT: right ventricular outflow; SP: spine; ST: stomach; SVC: superior vena cava; TA: tricuspid valve

According to the cardiac segment analysis method and the special anatomical structure characteristics of neonates, combined with domestic and foreign CHD screening standards and practical experience, our centre summarized this method and used it as the screening diagnostic standard. [8] This method includes 12 standard sections. There are six subxiphoid sections: the transverse section of the abdomen, the subxiphoid section of the dual atria, the four-chamber section, the left ventricular outflow tract section, the right ventricular outflow tract section, and the long axis view of the abdominal aorta. There is one apical region section: the apical four-chamber section. There are three parasternal sections: the long axis section of the left ventricle, a series of left ventricular short axis sections, and the short axis section of the cardiac base great arteries. There are two suprasternal fossa sections: the long-axis and short-axis views of the aortic arch (See Table 1; Fig. 1 for details.)

**Table 1:** Twelve-section ultrasonic screening and diagnosis method

probe position	section	figure	observation content
subxiphoid	transverse section of abdomen	Figure 1.1	the left and right atria were identified according to the positional relationship between viscera and great vessels.
	subxiphoid section of dual atria	Figure 1.2	observe the integrity of atrial septum and the return of vena cava and pulmonary vein
	four-chamber section	Figure 1.3	the ventricles were identified, and the orientation of cardiac axis, atrioventricular connection, integrity of atrial and ventricular septum, and pulmonary venous drainage were observed
	left ventricular outflow tract section	Figure 1.4	distinguish the aorta and pulmonary artery, clarify the connection between ventricle and great arteries, and observe whether there is outflow tract obstruction and ventricular septal defect
	right ventricular outflow tract section	Figure 1.5	
	long axis view of the abdominal aorta	Figure 1.6	it was used to observe whether there was a descending vertical vein, whether the inferior vena cava was interrupted, and whether there was stenosis and abnormal channels inferred by observing the spectrum of the abdominal aorta.
apical region	apical four-chamber section	Figure 1.7	Based on the standard four-chamber view, the beam was tilted cephalically until the left ventricular outflow tract was displayed, and tilted backward until the coronary sinus was visualized, so as to obtain a complete apical four chamber view
parasternal region	long axis section of left ventricular	Figure 1.8	They were mainly used to observe the connection between ventricle and great arteries and the spatial position of great arteries, conotruncal malformation, atrioventricular valve malformation, anomalous origin of coronary artery, aortopulmonary septal defect, anomalous origin of left and right pulmonary artery branches, ventricular septal defect, patent ductus artriosus, etc.
	a series of left ventricular short axis sections	Figure 1.9-1.11	
	short axis section of cardiac base great arteries	Figure 1.12	
suprasternal fossa	long axis section of aortic arch	Figure 1.13	They were mainly used for screening of congenital aortic arch anomalies. Such as: coarctation of aorta、interruption of aortic arch, double aortic arch, right aortic arch and vascular ring.
	short axis section of aortic arch	Figure 1.14	

#### Diagnosis of CHD and complex CHD

CHD was diagnosed according to the International Classification of Diseases code-10 (ICD-10),[9] excluding the following: (1) patent ductus arteriosus (PDA) < 3 months and (2) a patent foramen ovale or an atrial septal defect (ASD) ≤ 5 mm. The following conditions required dynamic monitoring: (1) Pulmonary artery branch stenosis: if the flow rate was less than 2 m/s and there was no local anatomical stenosis or dysplasia, follow-up could be conducted up to 3 months of age. A decrease in the flow rate to normal was considered physiological pulmonary artery branch stenosis. If the flow rate continued to increase, the patient was classified as having CHD. (2) When the flow rate of the aortic valve or pulmonary valve increased and there was no obvious abnormality in the structure or number of valve leaflets, if the cross-valve pressure gradient was less than 20 mmHg (1 mmHg = 0.133 kPa), follow-up could be conducted up to 3 months of age. If the pressure gradient was stable or showed a downward trend, follow-up observation was continued. If the pressure gradient did not decrease but increased, aortic or pulmonary artery stenosis was diagnosed. According to structure type, CHD was divided into simple CHD and complex CHD. Complex CHD refers to structural complex CHD excluding ASD, ventricular septal defect (VSD) and PDA.

#### Screening process

Eight sonographers from Tang Maternal and Child Health Care Hospital were trained by our centre, and all of them utilized the “twelve-section method” for screening after passing the examination. A PHILIPS iE33 ultrasound machine with S5-1 and S8-3 cardiac ultrasound probes was used. For the patients who screened positive, the centre regularly (every week) sent specialized staff to Tang County Maternal and Child Health Care Hospital for definite diagnosis (the local family members of the patients were contacted in advance). We recommended that newborns with negative screening results should be followed up for 1 year. In case of cardiac murmur, shortness of breath, cyanosis, recurrent pneumonia and no weight gain, we recommended that infants should come to our centre in a timely manner for a definite diagnosis.

#### Repeatability evaluation of the images obtained by trained physicians

Two trained sonographers were randomly selected to screen each newborn (with the informed consent of family members) via the twelve-section method, and the standard and clarity of the images were evaluated by two teachers with senior professional titles at our centre. The standard 12 sections were obtained by all the tested sonographers, and the repeatability was good.

#### Establishment of a hierarchical management system for CHD screening and treatment

**Fig. 2:**
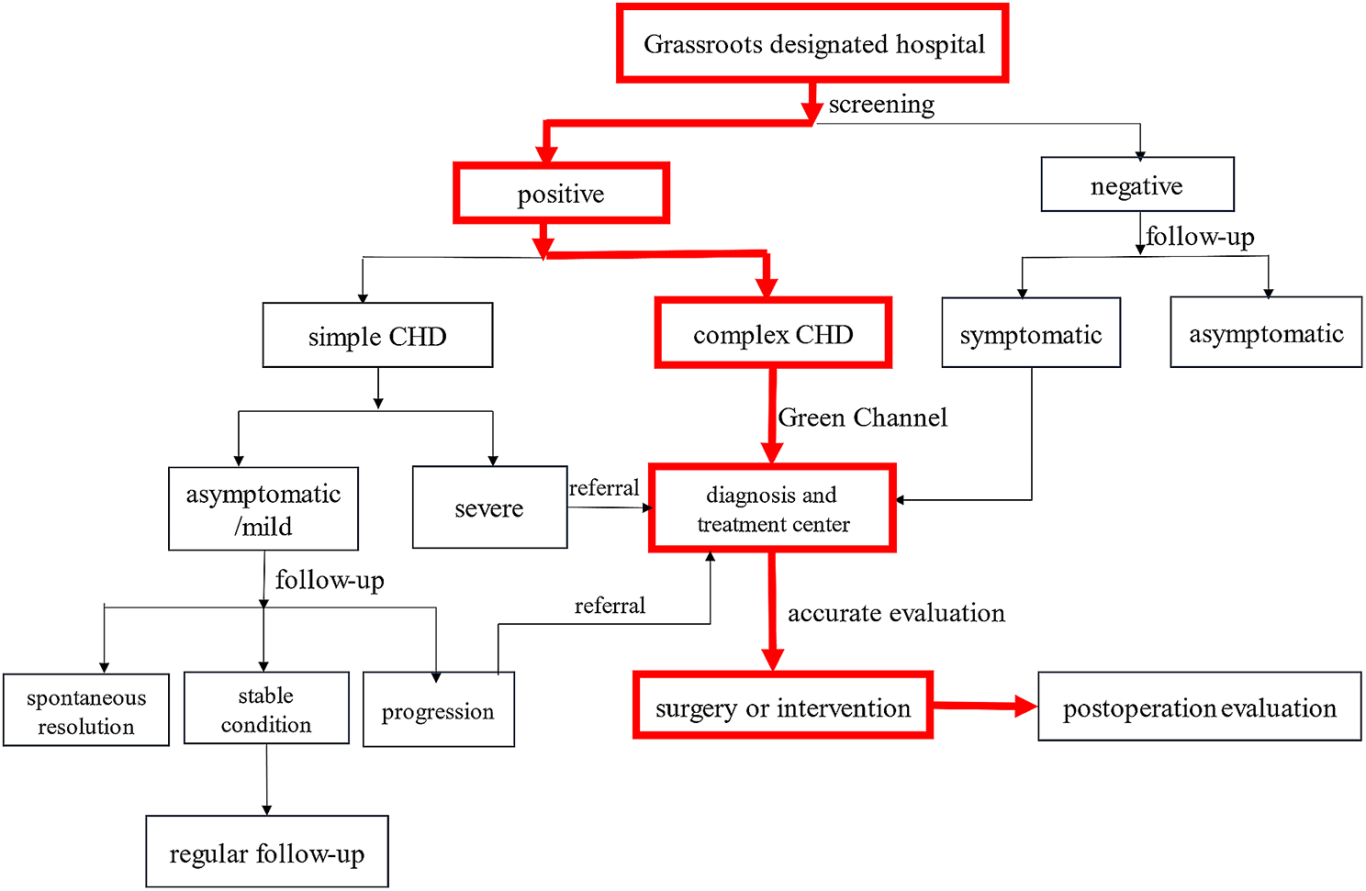
Hierarchical management system of CHD screening and treatment flow chart. This chart showed different levels of management according to the severity of congenital heart disease detected in newborns. CHD: congenital heart disease

Primary clinicians assessed the condition of patients with CHD, and patients with simple CHD with mild symptoms or no symptoms were reexamined locally. Patients with simple severe CHD and complex CHD were transferred to the centre for accurate diagnosis, evaluation and treatment, forming the hierarchical management system of CHD screening and treatment (See the flow chart of Fig. 2 for details).

#### Follow-up

Patients who were diagnosed with CHD or who required regular follow-up were followed up through outpatient visits, inpatient visits, or telephone calls until December 2021. The patients were followed up every 1–6 months according to their disease conditions.

### Sample size estimation and statistical analysis

We calculated the sample size based on the upper limit of CHD prevalence of 50‰; for an assumed error of 3‰, at least 20,275 consecutive newborns needed to be screened by echocardiography (using a two-sided α = 5%).

SPSS 23.0 statistical software was used for data analysis. The data were expressed as the mean ± standard deviation, median and percentage. Using the diagnostic results of our centre as the gold standard, the sensitivity, specificity and diagnostic coincidence rate of screening for complex CHD in Tang County were calculated. The t test was used for continuous variables, and the chi-square test or Fisher’s exact test was used for categorical variables. A probability value of *P* < 0.05 was considered to indicate statistical significance.

## Results

### Demographic characteristics

**Fig. 3:**
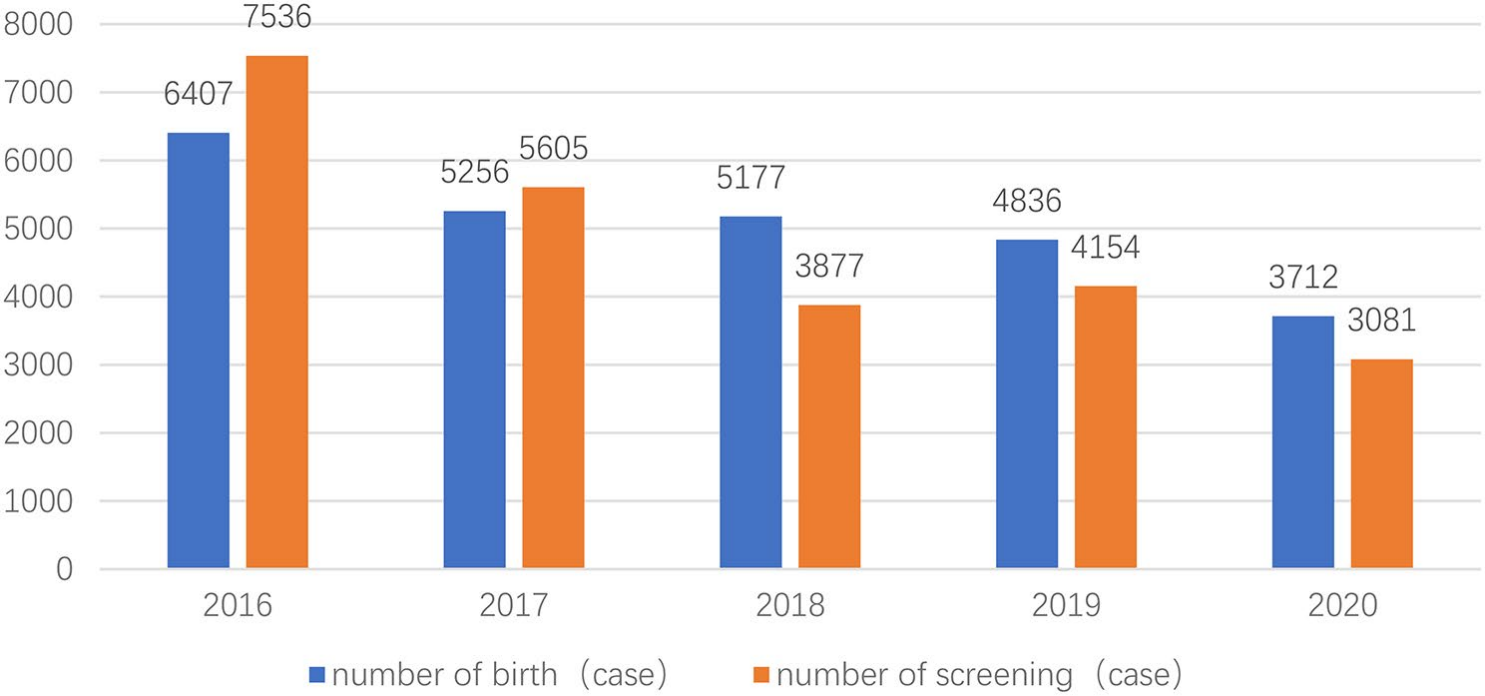
Screening coverage condition of congenital heart disease at live birth in Tang County. It showed the number of screening and the number of live births annually from 2016 to 2020. Note: This study was supported by National Energy Love Action Project. It requires as many regional neonatal CHD screening as possible to provide data for later work. Therefore, it consents that the grassroots implementation units can expand the screening scope to the surrounding areas. Therefore, the screening rate in 2016 and 2017 exceeded 100%

A total of 24,253 newborns were screened (25,388 born during the same period; the screening coverage rate was approximately 95.5%) (Fig. 3). There were 12,704 males (52.4%) and 11,549 females (47.6%). There were 2781 preterm infants (11.5%) and 21,472 term infants (88.5%). The mean birth weight was 3290 ± 490 g, the median gestational age was 38.5 weeks, and the median ultrasound screening time was 5 days.

### Simple and complex CHD screening via the “twelve-section method” in Tang County

A total of 301 infants with CHD, including 277 with simple CHD and 24 with complex CHD, were screened by the twelve-section method. Among the remaining 23,952 neonates with negative screening results, none sought medical treatment due to clinical symptoms during the 1-year follow-up. All 301 infants who screened positive were diagnosed at our centre, with 276 infants diagnosed with simple CHD and 25 diagnosed with complex CHD.

Screening at the grassroots level: The centre confirmed that, except for 2 cases of PDA missed in the diagnosis of VSD combined with severe pulmonary hypertension, other isolated or concomitant simple types of cardiac malformations were detected at the grassroots level. Among 277 cases of simple CHD diagnosed in Tang County, 2 cases of complex CHD were false negatives, including 1 case of total anomalous pulmonary venous connection (only diagnosed with the associated ASD and PDA) and 1 case of complete transposition of the great arteries (only diagnosed with the associated VSD and PDA). Among the 24 cases of complex CHD diagnosed by Tang County, the definite diagnosis by our centre showed that 1 case was a false positive (false echo loss of the aortopulmonary septum was misdiagnosed as an aortopulmonary septal defect), and 1 case was misdiagnosed as another complex CHD (Weinberg type B persistent fifth aortic arch coarctation was misdiagnosed as aortic coarctation). The sensitivity, specificity and coincidence rate of ultrasound for diagnosing complex CHD in Tang County were 92% (23/25), 99.6% (275/276) and 84% (21/24), respectively.

### Prevalence of CHD in Tang County

**Fig. 4:**
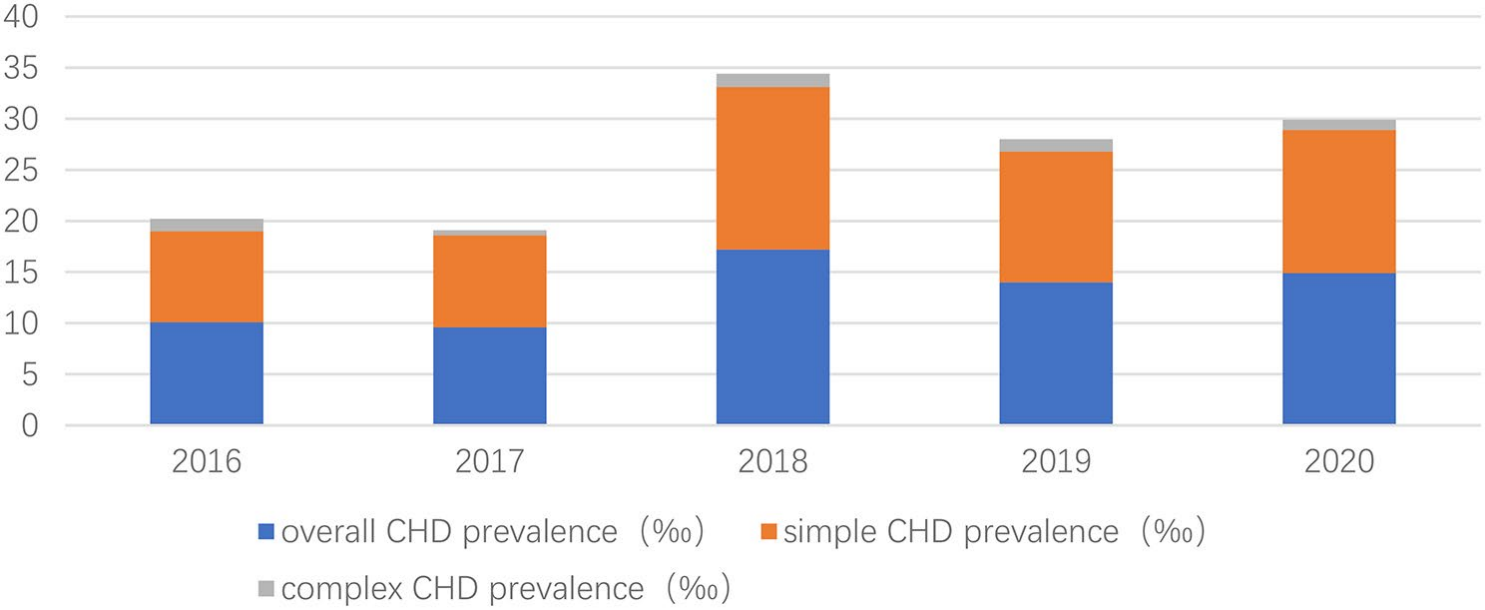
The prevalence of CHD in 5-year live births in Tang County. It presented that the trend of overall CHD prevalence in 5 years was consistent with that of simple CHD, with an upward trend, while the prevalence of complex CHD showed a stable state. CHD: congenital heart disease

The prevalence of overall CHD was 12.4‰. There were 128 males (42.5%) and 173 females (57.5%) with CHD. There were 276 patients with simple CHD, with a prevalence of 11.3‰. There were 25 patients with complex CHD, with a prevalence of approximately 1‰. Among them, VSD (159 patients, 6.6‰), ASD (128 patients, 5.3‰), PDA (33 patients, 1.4‰), total anomalous pulmonary venous connection (7 patients, 0.29‰), tetralogy of Fallot (3 patients, 0.12‰), double outlet right ventricular (3 patients, 0.12‰), complete transposition of the great arteries (3 patients, 0.12‰). 0.12‰) and atrioventricular septal defects (3 patients, 0.12‰) ranked in the top. There was only one case of left heart obstruction, which involved coarctation of the persistent fifth aortic arch (0.04‰) (Tables 2 and 3). The 5-year prevalence of overall CHD was 10.1‰, 9.6‰, 17.2‰,14‰, 14.9‰, respectively; the prevalence of simple CHD was 8.9‰, 9‰, 15.9‰, 12.8‰, 14‰, and the prevalence of complex CHD was 1.2‰, 0.5‰, 1.3‰, 1.2‰,1‰ (Fig. 4).

**Table 2:** Disease spectrum of congenital heart disease screened in Tang County

main malformations	total(case)	2016(case)	2017(case)	2018(case)	2019(case)	2020(case)
ASD	95	19	14	23	19	20
VSD	128	43	17	20	28	20
PDA	9	1	2	3	3	0
ASD+PDA	13	2	5	4	1	1
ASD+VSD	20	2	8	6	2	2
VSD+PDA	11	0	5	6	0	0
PS	2	0	1	1	0	0
PA	1	1	0	0	0	0
TA	2	1	0	1	0	0
TOF	3	1	0	0	2	0
DORV	3	0	0	2	0	1
TGA	3	0	1	1	0	1
TAPVC	7	5	1	0	1	0
Coarctation of PFAA	1	0	0	0	1	0
AVSD	3	1	0	0	1	1

ASD: atrial septal defect; AVSD: atrioventricular septal defect; COA: coarction of aorta; DORV: double outlet right ventricle; PA: pulmonary atresia; PFAA: persistent fifth aortic arch; PDA: patent ductus arteriosus; PS: pulmonary stenosis; TA: tricuspid atresia; TAPVC: total anomalous pulmonary venous connection; TGA: Complete transposition of the great arteries; TOF: tetralogy of fallot; VSD: ventricular septal defect

**Table 3:** Congenital heart disease spectrum details

Major deformity	type	number	detailed information
ASD	central	81	range 5–10 mm
	inferior vein cava	7	range 8–10 mm
	superior vein cava	5	range 5–6 mm
	mixed	2	range 10–15 mm
VSD	perimembranous	42	range 3–7 mm,5 cases with PH
	muscula	78	range 1–3 mm
	subarterial	8	range 4–8 mm,6 cases with PH
PDA	tubular	3	range 2–5 mm,1 case with PH
	conical	5	range of pulmonary artery end 2–4 mm, aortic end range about 4–6 mm, 2 cases with PH
	window	1	about 7 mm, with PH
ASD+PDA	13		ASD range 5–8 mm, ductus arteriosus range 3–4 mm, 1 case with PH
ASD+VSD	20		1case ASD 7 mm+VSD 12 mm, with PH; remaining ASD range 5–9 mm; VSD range 3–5 mm;5 cases with PH
VSD+PDA	11		VSD range 3–6 mm; ductus arteriosus range 3–5 mm;3 cases with PH
PS	severe	1	Without right ventricular dysplasia
	moderate	1	Without right ventricular dysplasia
PA	with VSD	1	Pulmonary artery was hypoplastic; PDA; collateral branches
TA		2	The relationship between aorta and pulmonary artery was normal, and both were with VSD and without PS
TOF		3	Pulmonary artery and its branches were hypoplastic in 2 cases; Pulmonary artery and its branches was well develpoed in 1 case
DORV		3	All were subaortic ventricular septal defect; infundibulum and pulmonary artery were well developed
TGA	complete	3	1 case was with VSD and PDA; 2 cases were with intact IVS and PDA
TAPVC	supracardiac	4	2 cases were without obstruction;2 cases were accompanied by obstruction, including 1 case with approximate atresia
	intracardiac	1	without obstruction
	subcardiac	2	Both 2 cases were accompanied by obstruction
Coarctation of PFAA	severe	1	The inner diameter of the narrowest part was about 2 mm
AVSD	partial	2	
	complete	1	

ASD: atrial septal defect; AVSD: atrioventricular septal defect; COA: coarction of aorta; DORV: double outlet right ventricle; PA: pulmonary atresia; PFAA: persistent fifth aortic arch; PDA: patent ductus arteriosus; PH: pulmonary hypertension; PS: pulmonary stenosis; TA: tricuspid atresia; TAPVC: total anomalous pulmonary venous connection; TGA: Complete transposition of the great arteries; TOF: tetralogy of fallot; VSD: ventricular septal defect

In terms of sex, the prevalence of overall CHD and simple CHD in females was significantly higher than that in males (*P* = 0.001). There was no significant difference in the prevalence of complex CHD between males and females. In terms of gestational age, preterm infants had a significantly higher prevalence of overall CHD and complex CHD than did full-term infants (*P* = 0.003). In terms of birth weight, the prevalence of overall CHD and complex CHD in low-birth-weight infants was significantly higher than that in normal-birth-weight infants (*P* = 0.004). The prevalence of CHD was higher in infants whose mothers were aged ≥ 35 years than in those whose mothers were aged < 35 years, but the difference was not significant (*P* > 0.05) (Table 4).

**Table 4:** Baseline clinical characteristics of newborns with CHD compared with those without CHD

Characteristics	Simple CHD(276)	complex CHD(25)	Total CHD(301)	Non-CHD(23,952)
Male/female	116/160*	12/13	128/173*	12,576/11,376
Preterm(<37 weeks)	36(13.1%)	5(20.5%)*	51(16.9%)*	2730(11.4%)
Birth weight (<2500 g)	20(7.2%)	5(20.5%)*	31(10.2%)*	1485(6.2%)
Maternal age(>35years)	15(5.3%)	2(6.8%)	17(5.5%)	1174(4.9%)

CHD: congenital heart disease; *indicates *p* < 0.05 (compared with newborns without CHD)

### Hierarchical management system for CHD screening and treatment and follow-up

All patients were included in the hierarchical management system for CHD screening and treatment. Among the 276 patients with simple CHD, 17 patients with severe CHD were referred directly to the centre for surgery. A total of 259 patients with asymptomatic or mild disease were followed up regularly in the local area. During the follow-up, 113 patients recovered spontaneously, 48 patients continued to be observed, 89 patients were referred to the centre for surgery when the disease progressed, 1 patient died without surgery, and 8 patients were lost to follow-up. Among the 25 patients with complex CHD, 18 patients were referred directly to the centre for surgery, 3 patients died without surgery, and 4 patients were lost to follow-up.

**Fig. 5:**
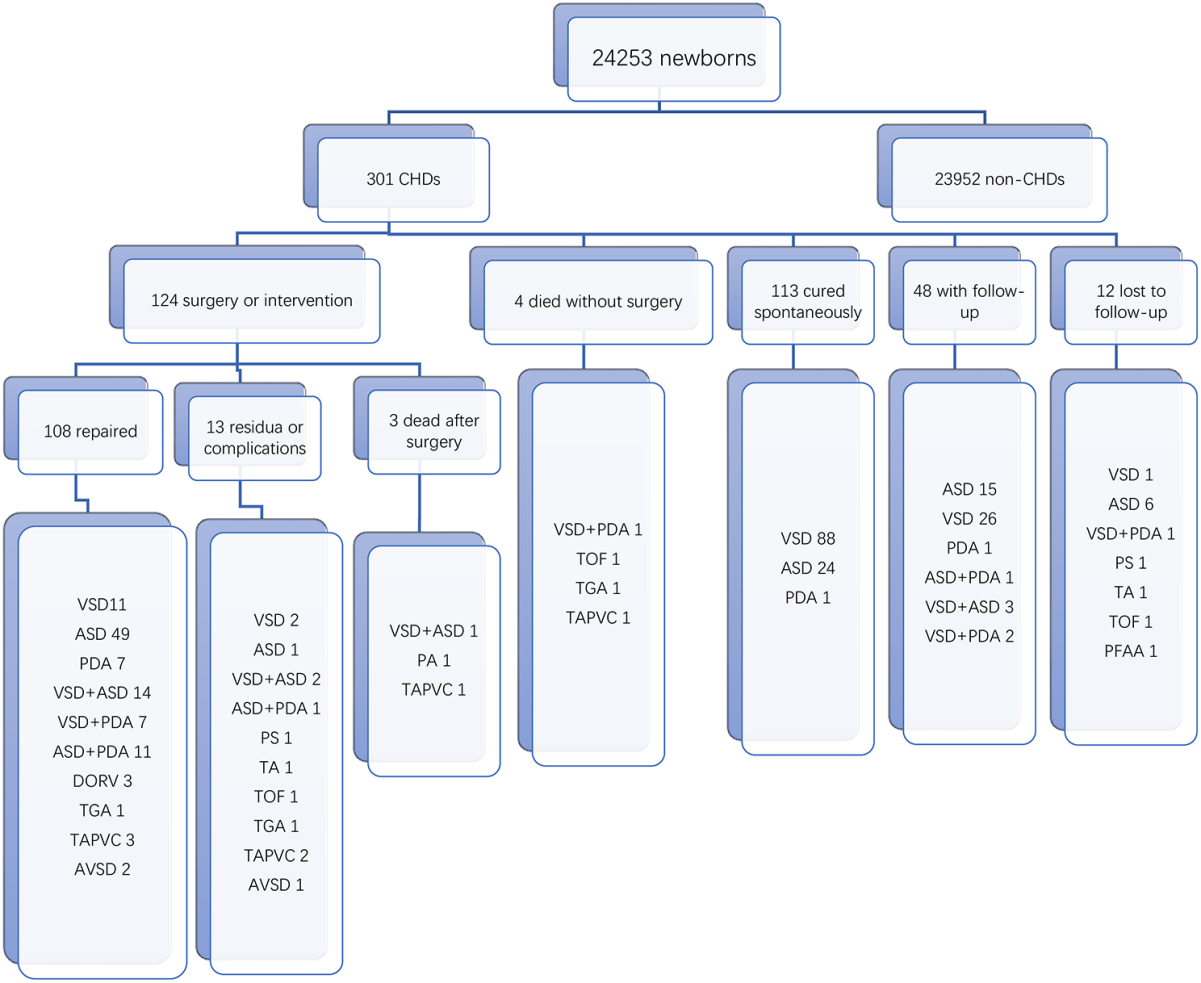
Follow-up results of newborns with congenital heart disease screened in Tang County until December 31, 2021. ASD: atrial septal defect; AVSD: atrioventricular septal defect; DORV: double outlet right ventricle; PA: pulmonary atresia; PFAA: persistent fifth aortic arch; PDA: patent ductus arteriosus; PS: pulmonary stenosis; TA: tricuspid atresia; TAPVC: total anomalous pulmonary venous connection; TGA: Complete transposition of the great arteries; TOF: tetralogy of Fallot; VSD: ventricular septal defect

Of the 301 patients with CHD, 113 (37.5%) recovered spontaneously. Eighty-eight patients with VSDs recovered spontaneously (55.3%, 88/159), of whom 66 (75%, 66/88) had muscular septal defects and 22 (25%, 22/88) had membranous septal defects. Spontaneous closure occurred in 34 patients (38.7%) before 6 months of age, 26 patients (29.5%) between 6 months and 1 year of age, and 28 patients (31.8%) after 1 year of age. Twenty-four patients (18.8%, 24/128) with ASD and 1 patient (9%, 1/33) with PDA recovered spontaneously. Forty-eight patients (15.9%) did not receive surgical treatment and continued to be observed. A total of 124 patients (41.2%) received surgery or interventional therapy. A total of 108 patients (87%) recovered well, 13 patients (10.5%) had postoperative complications or had residual shunts, and the long-term effects of these procedures need to be further evaluated. Three patients died after the operation (1 died of low cardiac output syndrome, 1 died of respiratory failure, and 1 died of pulmonary venous obstruction). Among the 4 patients who died without surgical intervention, 1 patient had VSD with PDA accompanied by trisomy 21 syndrome and intellectual impairment and died from a traffic accident during follow-up; 1 patient had severe tetralogy of Fallot complicated with neural tube malformation. Considering the poor prognosis, the family decided to stop treatment, and the child died after severe cyanosis during follow-up; 1 patient had complete transposition of the great arteries with VSD. The child had esophageal atresia type III, and died due to respiratory failure after severe pulmonary infection. One patient with complete anomalous pulmonary venous drainage (supracardiac type) and severe obstruction (approximate occlusion of the common pulmonary vein cava) died of severe hypoxia during transportation. The mortality rate for CHD was approximately 28.86 per 100,000 children, which was significantly lower than the average infant CHD mortality rate of 89.7 per 100,000 children in China. The case fatality rate of simple CHD was approximately 0.7% (2/276), and that of complex CHD was approximately 20% (5/25) (Fig. 5).

## Discussion

CHD is one of the most common congenital malformations. [1] There are many types of CHD, and the severity varies. Severe CHD, such as complex CHD with ductus arteriosus dependence (including pulmonary atresia with intact ventricular septum, complete transposition of great arteries with intact ventricular septum, and interruption of the aortic arch, et al.) requires immediate treatment after birth or during the neonatal period. In these newborns, the ductus arteriosus is an important channel of life, which is easily closed after birth, and improper oxygen inhalation will induce its closure. Prostaglandin E (intravenous drip) should be given immediately after early detection at the grassroots level to maintain the opening of the ductus arteriosus to buy time for subsequent transport and treatment. In addition, some complex and severe CHDs must be treated with emergency interventions, such as total anomalous pulmonary venous drainage with obstruction, during the neonatal period. [8] Therefore, performing ultrasonic screening and diagnosis at the grassroots level is highly important for hierarchical disease management, hierarchical diagnosis and treatment, scientific transportation, mortality reduction and surgical prognosis improvement. At present, the United States and other Western countries use pulse oximetry test, which has high specificity and sensitivity, to screen for severe neonatal CHD. [3–6] China uses the dual-index screening method (i.e., cardiac murmur plus pulse oximetry), which could improve the detection rate of severe neonatal CHD and has high specificity. [7] However, the above methods involve indirect clinical index interpretation, which couldn’t determine the type of CHD. Neonates with a positive dual-index screening result must undergo ultrasound examination for definite diagnosis. However, the use of ultrasound screening technology to screen for CHD in newborns at the grassroots level has not been standardized and has a low popularity rate and high rates of missed diagnosis and misdiagnosis. Primary ultrasound doctors cannot provide reliable ultrasound diagnoses for neonates who screen positive using the dual-index method. There are many sections of ultrasound accurate diagnosis method from diagnosis and treatment centres. Some of them are nonstandard sections, which are not easy to obtain, and it is difficult for grassroots physicians to master. Therefore, there is an urgent need for a reliable and practical ultrasonic screening method for neonatal CHD in primary hospitals, especially for complex and severe CHDs.

The acoustic windows under the subxiphoid position and suprasternal fossa are not often used for routine ultrasound examination of CHD in primary hospitals. [10] However, subxiphoid sections are irreplaceable views for the diagnosis of conotruncus malformations, and suprasternal fossa views are indispensable for the diagnosis of aortic arch abnormalities and other diseases. Therefore, routine examination methods in primary hospitals have great limitations. Following the cardiac segment analysis method, the subxiphoid and suprasternal fossa sections were added on the basis of the original examination sections used by the centre, and the “twelve-section method” was developed and used as the screening and diagnosis standard for CHD in primary medical institutions. A total of 301 CHD patients were identified in this study. Among the remaining 23,952 neonates who screened negative for CHD, none visited the hospital due to clinical symptoms during the 1-year follow-up, indicating that the false negative rate of the method is low. The centre confirmed that, except for 2 cases of PDA missed in the diagnosis of VSD combined with severe pulmonary hypertension, other isolated or concomitant simple types of cardiac malformations were detected at the grassroots level. The “twelve-section method” for complex CHD screening also had high sensitivity and specificity (92% and 99.6%, respectively), which fully reflects the reliability of this method and is conducive to the identification and treatment of patients with CHD, especially those with complex and severe CHD. In addition, among the final diagnoses of 25 patients with complex CHD, 8 had ductal-dependent CHD (1 patient with severe pulmonary stenosis, 1 patient with pulmonary atresia, 2 patients with tricuspid atresia, 3 patients with complete transposition of the great arteries, and 1 patient with severe coarctation of the persistent fifth aortic arch). Except for 1 patient with complete transposition of the great arteries who was missed due to poor sound transmission conditions under ventilator support and had a simple concurrent malformation, the remaining 7 patients with ductal-dependent CHD were detected by primary physicians by the “twelve-section method”. They were all immediately given Prostaglandin E (intravenous drip) to keep the ductus arteriosus open and were transferred to the centre for timely treatment (two patients were lost to follow-up in the later stage). Once again, this confirmed the reliability and feasibility of this method for screening and treatment at the grassroots level.

This study showed that the prevalence of CHD in Tang County was approximately 12.4‰, which was higher than the prevalence reported in the China Birth Defects Prevention report (4.095‰) in 2011. [11] The latter data were derived from birth defect registries, which were first based on the assessment of clinical manifestations. For those with negative clinical manifestations, it was inclined to miss diagnosis, so the prevalence was low. The trend of the 5-year prevalence of overall CHD was consistent with that of simple CHD, with an upward trend; however, the prevalence of complex CHD showed a stable trend, which again confirms the findings of Hoffman et al. that the prevalence of CHD largely depends on the prevalence of simple CHD, but the prevalence of complex CHD is relatively stable. [2]

In this study, there were more right ventricular outflow tract obstructive diseases (pulmonary stenosis, pulmonary atresia and tetralogy of Fallot) and fewer left ventricular obstructive diseases (aortic stenosis, coarctation of the aorta, interruption of the aortic arch and hypoplastic left heart syndrome) in China than in Western countries. [12, 13] The results of this study were similar to those of Huang GY et al., [14, 15] possibly because of genetic differences.

Several previous studies showed that men were more likely than women to have complex CHD, especially tetralogy of Fallot, complete transposition of the great arteries, and total anomalous pulmonary venous connection. [16, 17] There was no significant difference in the prevalence of complex CHD between males and females in this study. We could not exclude the possibility that prenatal screening for complex CHD resulted in pregnancy termination and affected the results. We found that preterm and low-birth-weight infants had higher prevalence of CHD than term and normal-birth-weight infants, which was consistent with the findings of previous studies. [17, 18] At present, the specific mechanism is unclear, and it may be that the presence of CHD in the foetal period affects foetal development and leads to premature birth and low birth weight. In addition, foetal CHD is likely to be associated with other organ malformations, which also tends to influence foetal development. This may also be related to the fact that infants with CHD are likely to also have chromosomal and genetic abnormalities. Previous studies have reported that the prevalence of CHD was significantly higher in infants whose mothers were aged ≥ 35 years than in those whose mothers were aged < 35 years. [19] However, there was no significant difference between the two groups in this study. For these reasons, pregnant women of advanced maternal age are a key focus group for prenatal care. Maternal health care and screening are conducive to the detection of complex CHD, and some pregnant women choose to terminate their pregnancy, which affects the outcome.

In this study, 301 patients were managed according to the hierarchical management of CHD screening and treatment. Infants with simple severe CHD (17 patients) and those with complex CHD (18 patients) were transported and received timely treatment after screening at the grassroots level. Subsequent follow-up showed that early intervention for children achieved good results, except for 3 cases of postoperative death, reflecting the positive significance of early screening at the grassroots level for timely treatment and prognosis improvement in patients with complex and severe CHD. During the local follow-up of children with simple asymptomatic or mild CHD, 113 patients recovered spontaneously. Forty-eight patients were stable and were observed locally. This approach greatly saved medical resources, avoided unnecessary long-distance transportation, and reduced the economic and time costs for families. Eighty-nine patients experienced progression during local follow-up, which mainly manifested as increased atrial or ventricular septal defects and widened ductus arteriosus; some of these patients experienced clinical symptoms such as left ventricular volume overload, recurrent pneumonia, heart failure, no weight gain, and pulmonary hypertension. All 89 patients were transferred to the centre in a timely manner for further diagnosis and treatment, which also reflected the close cooperation between the grassroots units and the diagnosis and treatment centre and accumulated valuable experience for further promoting the construction of a hierarchical management system for CHD screening and treatment. In addition, Shang WJ et al. reported that the CHD mortality rate of infants aged 0–1 years in China from 2004 to 2018 was 106.81 per 100,000 infants in 2004 and 38.70 per 100,000 infants in 2018. [20] Liu Z et al. reported the CHD mortality rate in children under 5 years old in China from 2010 to 2016, which was 155.0 per 100,000 children in 2010 and 89.7 per 100,000 children in 2016. [21] A total of 7 patients died in this study, with a mortality rate of 28.86 per 100,000 children, which was lower than the average infant CHD mortality rate in China. Previous studies have reported that if critically ill children with CHD were screened and diagnosed in time, the case fatality rate could be reduced to 20%, and the rehospitalization rate, length of stay and costs during hospitalization could be reduced. [22] In this study, the case fatality rate of severe and complex CHD was 14%, which was lower than the rate reported above, indicating that the application of the “twelve-section method” for screening at the grassroots level and the establishment of a hierarchical management system for CHD screening and treatment played a positive role in improving the screening and treatment of CHD and reducing the mortality rate of CHD, especially complex and severe CHD.

This was the first time that echocardiography was used to screen neonates for CHD in primary care units. The coverage was wide, and it truly reflected the prevalence of CHD in the primary areas. In addition, the “twelve-section method” for CHD screening was used in this study, and a hierarchical management system for CHD screening and treatment was established, which confirmed its feasibility and reliability in practice and provided a good approach for follow-up grassroots CHD screening and treatment. However, this study also had certain limitations. (1) The first limitation is that ultrasound is susceptible to interference from bone and air in the lungs and has certain limitations in the visualization of pulmonary arteriovenous fistula and vascular ring compression of the trachea. Moreover, due to the existence of physiological pulmonary hypertension in the neonatal period, when the abnormal coronary artery originates from the pulmonary artery, the flow of the coronary artery countercurrent into the pulmonary artery is not obvious, and the communication branches between the left and right coronary arteries are not abundant, so it is easy to make a missed diagnosis. The screening process should be combined with clinical manifestations and follow-up observation dynamically. If necessary, the diagnosis needs to be further confirmed by other imaging methods. (2) Prenatal termination of pregnancy and stillbirth were not included in the study. (3) This study did not include extracardiac malformations or genetic testing. We hope that we can further explore this topic in follow-up research.

## Conclusions

The “twelve-section method” is suitable for screening neonatal CHD at the grassroots level. The establishment of a hierarchical management system for CHD screening and treatment is conducive to the scientific management of CHD and has important clinical and social significance for the early detection, early intervention, reduction in mortality and improvement of the prognosis of complex and severe CHD.

## Data Availability

The datasets used and/or analyzed during the current study are available from the corresponding author on reasonable request.

## Abbreviations

CHD: Congenital heart disease
ASD: Atrial septal defect
VSD: Ventricular septal defect
PDA: Patent ductus arteriosus
Twelve-section method: Twelve-section ultrasonic screening and diagnosis method
